# Editorial Perspective: A plea for the sustained implementation of digital interventions for young people with mental health problems in the light of the COVID‐19 pandemic

**DOI:** 10.1111/jcpp.13317

**Published:** 2020-09-14

**Authors:** Michael Kaess, Markus Moessner, Julian Koenig, Sophia Lustig, Sabrina Bonnet, Katja Becker, Heike Eschenbeck, Christine Rummel‐Kluge, Rainer Thomasius, Stephanie Bauer, the ProHEAD Consortium

**Affiliations:** ^1^ Section for Translational Psychobiology in Child and Adolescent Psychiatry Department of Child and Adolescent Psychiatry University Hospital Heidelberg Heidelberg Germany; ^2^ University Hospital of Child and Adolescent Psychiatry and Psychotherapy University of Bern Bern Switzerland; ^3^ Center for Psychotherapy Research University Hospital Heidelberg Heidelberg Germany; ^4^ Section for Experimental Child and Adolescent Psychiatry Department of Child and Adolescent Psychiatry University Hospital Heidelberg Heidelberg Germany; ^5^ Department of Child and Adolescent Psychiatry, Psychosomatics and Psychotherapy Philipps‐University of Marburg Marburg Germany; ^6^ Marburg Center for Mind, Brain and Behavior (MCMBB) Philipps‐University of Marburg Marburg Germany; ^7^ Department of Psychology University of Education Schwäbisch Gmünd Schwäbisch Gmünd Germany; ^8^ Department of Psychiatry and Psychotherapy University Leipzig Leipzig Germany; ^9^ German Center for Addiction Research in Childhood and Adolescence University Hospital Hamburg‐Eppendorf Hamburg Germany

## Abstract

The coronavirus disease 2019 pandemic and the consequent global lockdown posed a particular challenge for youths with mental health problems. Crucial interference with their everyday lives likely increased psychological distress while accessibility of conventional mental health care was limited. Ongoing online trials offer a unique opportunity to analyse mental health status and help‐seeking behaviour of adolescents during the pandemic. The ProHEAD‐online trial aims at improving help‐seeking behaviour of children and adolescents with significant psychological impairment. From January to May 2020, 1,042 students had access to the ProHEAD‐online platform providing information on mental illness, monitoring, peer support and professional counselling. In the week from 11 March, when schools were closed in Germany, a drastic (more than 2 standard deviations) but time‐limited increase in utilization of the ProHEAD‐online services became apparent. This may indicate a worsened mental health status and an increased help seeking via digital services during the lockdown. Although this finding is purely observational, it speaks to the importance of evidence‐based online service in the field of mental health within the current crisis and beyond.

Numerous commentary and viewpoint papers have recently discussed the expected direct and indirect economic, social and psychological effects of the coronavirus disease 2019 (COVID‐19) pandemic including the related global lockdown. Children and adolescents around the world were and still are affected by containment efforts such as school closings, stay‐at‐home orders and physical distancing which have led to abrupt and significant changes in their everyday lives and social relationships. While the exact impact of the current crisis and the various pandemic‐control decisions on the mental health and well‐being of young people is largely unknown, experts assume a significant increase in the burden of mental illness, and particular concerns have been raised about the worsening of pre‐existing mental health problems, discontinuity of care and an exacerbation of the already pervasive challenge of unmet treatment needs among young people (Fegert, Vitiello, Plener, & Clemens, 2020; Loades et al., 2020).

Since the outbreak of the pandemic has considerably disrupted the delivery of mental healthcare (e.g. reduction or unavailability of in‐person visits for outpatient psychiatric treatment or psychotherapy), young people may face even more barriers to professional care than in prepandemic times. In response to the outbreak, and in line with the World Health Organization's timely strategic call to ‘reinforce or establish web‐based and other telemedicine platforms to provide direct clinical services and provide clinical decision support’ (World Health Organization, 2020, p. 4), professional organizations and regulatory bodies in various countries have revised policies and regulations and encouraged providers to use digital interventions to support patients with mental illness. Recently, it was even argued that telemedicine may have the potential to lower the barriers to seeking treatment for young people (Hoekstra, 2020).

Obviously, the sudden onset of the COVID‐19 pandemic did not allow for the initiation of rigorous studies specifically investigating the immediate effects of the lockdown on the psychological health of young people, their help‐seeking behaviour or their utilization of digital mental health interventions. However, ongoing trials offer unique opportunities to provide insights into these issues and inform future research, intervention development and service delivery beyond the current pandemic. To that end, we recently analysed utilization data of a digital intervention for young people with mental health problems, that is ‘ProHEAD‐online’ (‘Promoting Help‐seeking using E‐Technology for ADolescents’; Kaess et al., 2019). ProHEAD‐online addresses high school students aged 12 to 19 years who report significant psychological impairments (including suicidality) and do not currently receive professional treatment. The intervention aims at improving participants’ help‐seeking behaviours and at facilitating access to professional mental healthcare and includes the following modules: Tailored psychoeducational materials provide information on mental health problems in young people, professional health care and contact information of regional providers. Via an automated monitoring system, participants are asked about their help‐seeking behaviour, treatment uptake and potential barriers on a regular basis. In addition, they are invited to communicate with peers via a messaging system or online chat. Finally, they may use professional counselling via online messaging, telephone or chat. The efficacy and cost‐effectiveness of the intervention are currently being studied in a two‐arm randomized clinical trial (target sample size: 1,500 participants) which is part of a large collaborative initiative on e‐mental health for young people funded by the German Ministry of Education and Research (Kaess & Bauer, 2019). While the intervention group has access to all modules of ProHEAD‐online, participants in the control group may only access limited content.

For the purpose of this editorial perspective, we report and discuss participants’ utilization of ProHEAD‐online over a 4‐month period (1 January 1 to 5 May 2020). We created a composite measure of ProHEAD‐online utilization including number of logins, clicks and chat participation aggregated by week (standardized: *M* = 0, *SD* = 1). Since the start of the ProHEAD consortium, 5,447 students from 77 schools at five study sites were screened for their mental health. Out of these, 1,143 students reported substantial mental health problems and were consequently eligible for participation in ProHEAD‐online. Participants were included in the trial independent of their current treatment status. While the automated monitoring of help‐seeking behaviours, treatment uptake and barriers in the intervention group was stopped as soon as participants reported that they took up treatment, they could still login to ProHEAD‐online and utilize the other modules. Between January and May 2020, we had 1,042 students (72% female; mean age 14.6 years) having access granted to the full ProHEAD‐online intervention (intervention group) or the limited version (control group). Among those, we observed a substantial, but clearly time‐limited increase in utilization of ProHEAD‐online (increase of more than 2 *SD* of the composite measure) starting in the week of 11th of March (Figure S1). Within this week, the nation‐wide closing of schools in Germany was announced and executed, and physical distancing regulations were implemented. After three weeks, utilization of the intervention went back to an average level comparable to the prepandemic user activity. This finding may indicate that a subgroup of participants experienced a deterioration of their mental health status which led to an increased need for professional help and thus an increased utilization of the digital intervention. Also, the observed pattern may reflect participants’ uncertainties related to the limited availability and accessibility of conventional mental health care during the lockdown which led them to utilize the digital intervention more than usual. In addition, our finding highlights the potential that established and already disseminated digital interventions might have had during the COVID‐19 pandemic, both as a direct source of mental health support and as a means of reducing disruption to ongoing intervention normally provided face to face.

While we acknowledge that this finding is purely observational, we believe it clearly speaks to the potential value of a sustained implementation of digital mental health interventions into routine care, that is, evidence‐based tools that can be easily accessed and made available on a permanent basis, so that young people can turn to them immediately in times of need. We anticipate and hope that reports and empirical data on the uptake, utilization and outcome of other digital interventions during the present crisis along with reports on the experiences of practitioners and patients may contribute to an enhanced and lasting integration of e‐mental health and conventional mental healthcare. Despite a solid evidence base on the efficacy of digital interventions, this integration has not progressed fast enough over the past decade, mostly due to structural barriers, reluctance of stakeholders and a lack of capacity building, acceptance and training among clinicians. Thus, in many countries, the potential of e‐mental health remained largely untapped so far. However, in the light of the COVID‐19 pandemic, there seems to be broad consensus concerning the usefulness of and the need for evidence‐based digital interventions (e.g. Torous, Myrick, Rauseo‐Ricupero, & Firth, 2020; Wind, Rijkeboer, Andersson, & Riper, 2020).

There is no universal model how digital approaches may enhance care for mental illness. Obviously, the availability, accessibility and affordability of conventional health care, which all depend substantially on the specifics of national healthcare systems, play a key role. In countries with few professional treatment providers or poor health insurance coverage, Internet‐based, mobile self‐management interventions or therapeutic apps may be a valuable resource to support otherwise underserved populations. When therapeutic resources are less scarce, remote treatment via videoconferencing or online chat may be a viable, cost‐neutral alternative to face‐to‐face care. However, in many service systems, the biggest potential of e‐mental health tools may actually lie in the opportunity to improve help seeking and access to professional treatment (as described for the example of ProHEAD above) as well as in two other options: On the one hand, digital approaches allow us to significantly improve the continuity of care, for example in the context of stepped care approaches where online tools are used to provide pre‐ or post‐treatment support or in the context of the management of chronic mental illness where technology‐enhanced low‐threshold monitoring allows for the immediate detection and intervention of recurrent illness episodes. On the other hand, blended treatment approaches, that is the utilization of digital tools in parallel to face‐to‐face treatment, appear highly promising. Blended care either aims at improving treatment efficacy (when digital interventions are used as add‐on to conventional care) or at improving treatment efficiency (when part of the treatment is delivered via technology and the costly time in the face‐to‐face setting is thus reduced).

Future research and experiences from clinical practice will help to identify the best ways how to harness digital approaches in various care settings, service systems and during unforeseeable circumstances such as the COVID‐19 pandemic. In that sense, the present crisis actually offers promising opportunities for our field to work towards enhanced models of service delivery in order to mitigate the detrimental impact of mental illness on the lives of young people.

## Supporting information

**Figure S1.** Utilization of ProHEAD‐online over time (composite measure including number of logins, clicks, and chat participation aggregated by week; standardized: *M* = 0, *SD* = 1). Dates represent the first day of the respective week.

## ProHEAD Consortium

Michael Kaess (PI), Stephanie Bauer (PI), Markus Moessner, Julian Koenig, Sabrina Bonnet, Stella Hammon, Sophia Lustig, Regina Richter, Katja Bertsch, Romuald Brunner, Johannes Feldhege, Christina Gallinat, Peter Parzer, Johanna Sander (University Hospital Heidelberg, Heidelberg, Germany). Rainer Thomasius (PI), Silke Diestelkamp, Anna‐Lena Schulz, Tabea Theissen (University Hospital Hamburg‐Eppendorf, Hamburg, Germany). Christine Rummel‐Kluge (PI), Sabrina Baldofski, Elisabeth Kohls, Lina‐Jolien Peter, Mandy Rogalla, Sarah‐Lena Klemm (University Hospital Leipzig, Leipzig, Germany). Heike Eschenbeck (PI), Vera Gillé, Laya Lehner, Hanna Hofmann (University of Education Schwäbisch Gmünd, Schwäbisch Gmünd, Germany). Katja Becker (PI), Alisa Hiery (Philipps‐University of Marburg, Marburg, Germany). Hans Joachim Salize (PI), Dr. Elke Voss (Central Institute of Mental Health Mannheim, Mannheim, Germany). Dr. Steffen Luntz (Coordination Center for Clinical Studies (KKS), Heidelberg, Germany).
