# Supplementary material for: Editorial Perspective: A plea for the sustained implementation of digital interventions for young people with mental health problems in the light of the COVID‐19 pandemic
Source: J Child Psychol Psychiatry. 2020 Sep 14;62(7):916–8. doi: 10.1111/jcpp.13317 (PMC8359353; doi:10.1111/jcpp.13317)
Supplement: Supplementary file 1 — Figure S1. Utilization of ProHEAD‐online over time (composite measure including number of logins, clicks, and chat participation aggregated by week; standardized: M = 0, SD = 1). Dates represent the first day of the respective week. [file JCPP-62-916-s001.docx]

**Figure S1.** Utilization of ProHEAD-online over time (composite measure including number of logins, clicks, and chat participation aggregated by week; standardized: M=0, SD=1). Dates represent the first day of the respective week.
